# PSC-db: A Structured and Searchable 3D-Database for Plant Secondary Compounds

**DOI:** 10.3390/molecules26041124

**Published:** 2021-02-20

**Authors:** Alejandro Valdés-Jiménez, Carlos Peña-Varas, Paola Borrego-Muñoz, Lily Arrue, Melissa Alegría-Arcos, Hussam Nour-Eldin, Ingo Dreyer, Gabriel Nuñez-Vivanco, David Ramírez

**Affiliations:** 1 Center for Bioinformatics, Simulations, and Modeling (CBSM), Faculty of Engineering, University of Talca, Talca 3460000, Chile; avaldes@utalca.cl (A.V.-J.); idreyer@utalca.cl (I.D.); ganunez@utalca.cl (G.N.-V.); 2 Instituto de Ciencias Biomédicas, Universidad Autónoma de Chile, Santiago 8900000, Chile; carlos.pena01@uautonoma.cl (C.P.-V.); lily.arrue@cloud.uautonoma.cl (L.A.); melissa.alegria@uautonoma.cl (M.A.-A.); 3 Bioorganic Chemistry Laboratory, Facultad de Ciencias Básicas y Aplicadas, Campus Nueva Granada, Universidad Militar Nueva Granada, Cajicá 250247, Colombia; u7700101@unimilitar.edu.co; 4 DynaMo Center, Department of Plant and Environmental Sciences, University of Copenhagen, 1017 Copenhagen, Denmark; huha@plen.ku.dk

**Keywords:** plant secondary metabolites, natural products, 3D-structures, chemical and structural properties, physicochemical and pharmaceutical descriptors, database

## Abstract

Plants synthesize a large number of natural products, many of which are bioactive and have practical values as well as commercial potential. To explore this vast structural diversity, we present PSC-db, a unique plant metabolite database aimed to categorize the diverse phytochemical space by providing 3D-structural information along with physicochemical and pharmaceutical properties of the most relevant natural products. PSC-db may be utilized, for example, in qualitative estimation of biological activities (Quantitative Structure-Activity Relationship, QSAR) or massive docking campaigns to identify new bioactive compounds, as well as potential binding sites in target proteins. PSC-db has been implemented using the open-source PostgreSQL database platform where all compounds with their complementary and calculated information (classification, redundant names, unique IDs, physicochemical properties, etc.) were hierarchically organized. The source organism for each compound, as well as its biological activities against protein targets, cell lines and different organism were also included. PSC-db is freely available for public use and is hosted at the Universidad de Talca.

## 1. Introduction

Plants produce a vast and diverse assortment of organic compounds, the majority of which do not appear to participate directly in growth and development. These substrates, traditionally referred to as Plant Secondary Compounds (PSC), are often differentially distributed among limited taxonomic groups within the plant kingdom [1] and make major contributions to the specific colors, tastes and odors, as well as providing defense properties. For humans, one of the most important applications of plant secondary compounds is their use as traditional medicines and pharmaceuticals [2]. For example, a positive correlation has been demonstrated between the bioactive molecules of green tea and the prevention of obesity or the protection of the cardiovascular system [3,4]. Compounds from *Cannabis sativa*, such as phytocannabinoids, are used as therapeutic tools in cancer [5], central nervous system disorders [6], epilepsy [7], and other pathologies. In addition, phenolic compounds such a resveratrol, present in grapes and wine [8], have shown neuroprotective properties [9]. 

More than 100 natural product databases have been created in the last 20 years [10]. For example, SuperNatural II [11], The Universal Natural Products Database [12], NP Atlas [13] ZINC [14], NPASS [15], BindingDB [16], NPEdia [17], 3DMET [18], and the CNPD [19] are some of the most popular and extensive ones. Unfortunately, several of them are no longer maintained. Others are designed only as a repository of chemical compounds and the majority of the databases do not allow for the selection, grouping, and downloading of all the structural and physicochemical features of a particular subset of compounds. Also, in other cases, there is no link to related or original databases. Without these characteristics and considering the large number of molecules that are currently registered in public databases, a given repository of biological information might not be useful. In addition, some databases include experimental and computer-based characteristics of the stored compounds, but they often do not consider additional analyses or correlations between the data [10]. Remarkable, our database allows finding molecules by name or structure, to group and bulk downloading of properties and structures. Moreover, all compounds are linked to the original sources and for most cases, several new physicochemical features were calculated and are available. It is also worth pointing out that the conventional discovery of new bioactive compounds from natural resources required laborious screening of several compound libraries. However, with increasing insights into the structure function relationship of target proteins, today it is possible to screen virtually those large chemical libraries, as well as subsets of known compounds to predict new activities. Although a large number of databases have been created over the years, it is currently not possible to efficiently extract delimited 3D-structural information along with physicochemical and pharmaceutical ones of a given group of plant secondary compounds needed to perform such screens.

In this context, and in the interest of studying phytochemical compounds with potential application in drug discovery processes by computational tools, we present PSC-db, a web-based searchable database of Plant Secondary Compounds (PSC). PSC-db incorporates a user-friendly search tool which allows us to find compounds according to their chemical classification and 3D-structural similarity using the Tanimoto coefficient parameter [20]. Search-filters can be applied such as compound PSC-db ID, name, molecular formula, molecular weight or using certain descriptors such as the number of carbon atoms in a given group of pre-selected compounds. All these filtered data, containing the 3D structure of the compound and its computed features, can be downloaded as SDF and CSV format files, respectively. Additionally, PSC-db allows for computing the correlation between any pair of the available features among a subset of compounds (physicochemical properties, lipophilicity, water solubility, pharmacokinetics and drug-likeness). Interestingly, these types of correlations have been reported, for example, to validate the estimations of the permeability of compounds [21], the estimations of oral absorption [22], and to compare the ADME/Tox profiling of several important chemical compound databases [23]. Nowadays, it is useful to have a repository that helps the users to find natural compounds with potentially similar properties/bioactivities. For example, a database that stores thousands of molecules and also performs a clustering based on physicochemical features or drug-like properties, could be useful in the identification or possible new uses of known drugs. In fact, our understanding of how these active compounds exert their bioactivity could be boosted by the comprehension of how known molecules belong to the same cluster. Thus, PSC-db can help to identify compounds that have never been tested, accelerating the processes of bioactive identification. 

PSC-db is unique in providing this bundled information and thus distinguishes itself from other databases. A new database offers the opportunity to reduce the amount of resources and time in the rational drug design processes, in which PSC are used as bioactive compounds or as pharmaceutical excipients. This is why we expect that the new structural, physicochemical, and pharmaceutical insights provided by PSC-db will significantly contribute to the ongoing global efforts to develop novel products, as well as to expand the range of applications based on these PSC.

## 2. Results

**Figure 1:**
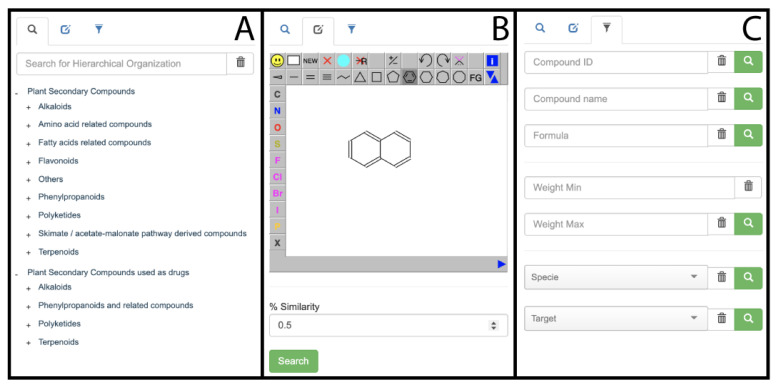
PSC-db search function. Users can search by the phytochemical compounds’ classification (**A**), by structural similarity (**B**), and by the following filters: compound ID, name, molecular formula, molecular weight range, source organism (species) and biological target (**C**).

Here, we present PSC-db, a web-based searchable database of 2853 Plant Secondary Compounds (PSC) hosted at the Universidad de Talca. The database is freely available for public use at http://pscdb.appsbio.utalca.cl (accessed on 6 January 2021). PSC are classified into 9 subfamilies according to their phytochemical properties and chemical composition: (1) alkaloids, (2) amino acid-related compounds, (3) fatty acids related compounds, (4) flavonoids, (5) phenylpropanoids, (6) polyketides, (7) skimate/acetate-malonate pathway derived compounds, (8) terpenoids, and (9) others. Due to the relevance of PSC for the development of therapeutic agents with pharmacological activity [24], we present a subset of 305 compounds from the PSC-db, which are approved to be used as drugs. We implemented a user-friendly alternative that allows to search for compounds according to their chemical classification, 3D-structural similarity using the Tanimoto coefficient parameter, or also applying filters such as compound PSC-db ID, name, molecular formula, molecular weight, source organism (specie) or biological target (Figure 1).

**Figure 2:**
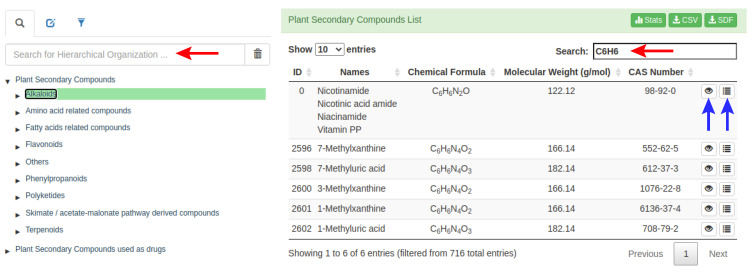
Selection of multiple compounds using two different searching tools (red arrows). For each phytochemical compound the PSC-db ID is given, as well as the names, chemical formula, molecular weight, and CAS number (right panel). More detailed information is available through the “eye” and “list” buttons (blue arrows).

Additionally, the user can apply a second search filter within a set of compounds, using certain descriptors, such as the number of carbon atoms in a given group of pre-selected compounds. Figure 2 presents an example where all the alkaloids were pre-selected (Figure 2, left red arrow), and then a second filter was applied to select the PSC with six carbons and six hydrogens “C6H6” (Figure 2, right red arrow). As a result, 716 alkaloids were pre-selected with the first filter, and then six compounds were obtained following the second filter (Figure 2). After the final selection of the phytochemical compounds, information such as the PSC-db ID, names, chemical formula, molecular weight, and CAS number is displayed in a dynamic table, and two buttons allow the user to access more detailed information for each compound (Figure 2, blue arrows). The “eye” button allows access to the 2D- and 3D-structure, as well as a Bioavailability RADAR, calculated from the predicted ADME/Tox properties (Figure S1). The 3D representation is interactive, and the structure can be rotated to fit the user’s needs. The “list” button allows access to more detailed information on each compound such as external links associated with other databases (NIKKAJI, KNApSAcK, PubChem, KEGG, ChEMBL, and ZINC). This might be very useful. For instance, to know if one or more compound(s) are commercially available, it is possible to access the ZINC link to find the different vendors and the availability of the compound. The hierarchical organization is also indicated (Figure S2), as well as the predicted physicochemical and pharmaceutical properties calculated using SwissADME server [25] (Figure S3). Finally, the user can access to the source organism for a given phytochemical compound, as well as to its biological activity information (Figure S4). The source organism and biological activity information was retrieved from KNApSAcK and ChEMBL databases, respectively. 

The 3D-structures provided for each phytochemical compound (“eye” button in Figure 2) were obtained from PubChem database [26]. The 3D structures were generated with OEOmega (OEOmega, version 2.4. OpenEye Scientific Software, Inc.; Santa Fe, NM, USA: 2009), only for compounds that fulfill the following criteria: Not too large (≤50 non-hydrogen atoms), not too flexible (≤15 rotatable bonds), consists of only organic elements (H, C, N, O, F, Si, P, S, Cl, Br, and I), has a single covalent unit (i.e., not a salt or a mixture), and contains only atom types recognized by the MMFF94s force field [27,28]. Each computed 3D conformer is not at an energy minimum and may not represent the lowest energetic form in vacuum, solvent, or a binding pocket. Therefore, it is recommended to minimize the energy of the phytochemical compounds before using them in molecular simulations. The PSC-db 3D-structures presented here describe energetically-accessible and (potentially) biologically relevant conformations of a phytochemical structure.

**Figure 3:**
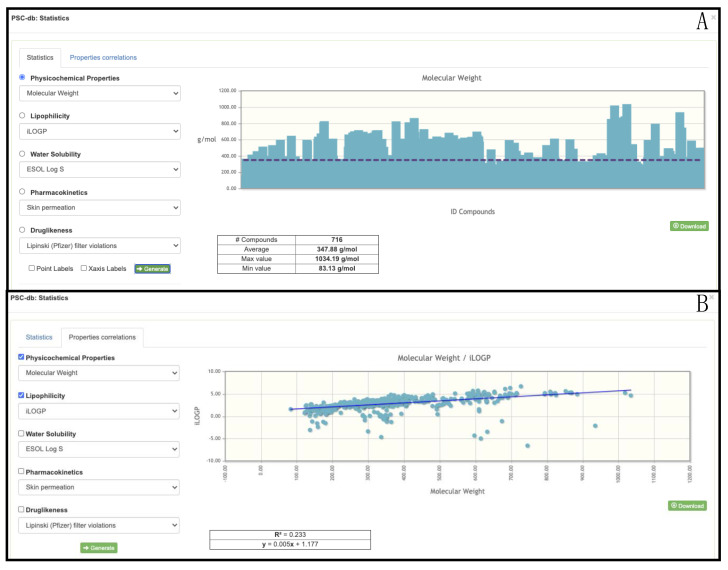
PSC statistics based on the calculated physicochemical and pharmaceutical properties. Statistics for the molecular weight (**A**) and the correlation between the molecular weight and the lipophilicity (**B**) of 716 selected alkaloids are shown.

Finally, we have provided a tool to calculate and to plot different statistics using the predicted physicochemical and pharmaceutical properties (Figure S3). To access this section the user should use the “Stats” button (Figure 2), which will provide access to the *statistics* and *properties correlations* options. The *statistics* option allows to graph one of the selected properties for a chosen group of compounds. In Figure 3A the molecular weight (MW) of 716 alkaloids (preselected compounds) is shown as a bar chart, and the average, min, and max MW values are given. In Figure 3B the correlation between the MW and the lipophilicity of the group of the same compounds is displayed. With this feature, the user is able to correlate different families by their physicochemical and pharmaceutical properties. For instance, alkaloids and flavonoids could be correlated concerning their molecular weight, the Topological Polar Surface Area (TPSA), or with respect to properties such as lipophilicity, water-solubility, or drug-likeness.

## 3. Discussion

PSC-db provides the key information of several phytochemical compounds; we categorized 2853 Plant Secondary Compounds (PSC) into 9 subfamilies according to their phytochemical properties and chemical composition. We computed relevant physicochemical characteristics for all PSC and then significant pharmaceutical properties were calculated to estimate the ADME (absorption, distribution, metabolism, and elimination) profiles of the PSC. The information about source organism for each PSC, as well as its biological activity against proteins and cell lines was also integrated in the database.

In the PSC-db, among the basic functionalities, we incorporated several features not included in other databases, such as advanced search filters by name and alternative names, by molecular weight, by 3D-structure similarity using the Tanimoto coefficient [29], and also by source organism and biological target. So, if the user would like to know, for example, in which species quercetin can be found (a flavonol that is found in many fruits, vegetables, leaves, seeds, and grains), through the “list” button (Figure 2) the user will find that quercetin is present in more than 140 different plant species, and also in *Mucor hiemalis*, from the Fungi kingdom. If otherwise, the interest is focused on the biological activity of the phytocompound, and to know particularly what biological assays have been carried out (on proteins, cell lines, organisms, ADMETox assays, etc.) through the information contained in PSC-db; the user will be able to access this data easily, together with the respective reference where the activity is reported.

A 3D-molecular visualization by using 3Dmol.js was also incorporated with the full implementation of the entire set of functionalities [30]. The 3D structures of the phytochemical compounds deposited in the PSC-db can be downloaded individually (MOL2 format) or in groups (SDF format), depending on the selection made by the user through the multiple filters available in the database. Along with this 3D-structure download, the physicochemical and pharmaceutical properties of the selected phytochemical compounds can also be downloaded in a CSV file (Figure 2, *CSV button*). If the entire data deposited in the PSC-db is needed, a button to access and download all the data (in different formats) is available.

Among the possible applications of our database is, for example, the search for PSC with some similarity to a molecule of interest. For instance, if the user wants to know which PSC have structure similarity to the neurotransmitter serotonin, and therefore have similar biological and pharmacological properties, and can potentially be used as new drugs, by using the structure similarity search tool (Figure 1B) it is possible to identify 10 phytocompounds when a 0.5 of % similarity threshold is set. Among the identified compounds, tryptamine and tetrahydroharmine can be found. Tryptamine is a monoamine alkaloid that can be used as a non-selective serotonin receptor agonist and serotonin-norepinephrine-dopamine releasing agent, with a preference for evoking serotonin and dopamine release over norepinephrine release [31,32]. Tetrahydroharmine is a fluorescent indole alkaloid that weakly inhibits serotonin re-uptake and may be used as antidepressant agent [33].

One of the biggest challenges we dealt with is to collect, group and classify data from the vast universe of PSC (>200,000). Currently, there is no repository that can effectively present all known PSC, and the reason is that classifying and characterizing them is not an easy task and involves the optimization of data science, as well as artificial intelligence algorithms tailored for this group of chemical compounds. For this reason, we expect to constantly update the PSC-db with the information deposited in other databases such as PubChem and ChEMBL, manually curating the PSC and calculating their physicochemical properties in order to categorize them according to the system established in our repository, including alternative names, 2D- and 3D-structures, external links, hierarchical organization, physicochemical and pharmaceutical properties (ADME profile), source organism and biological activity. 

The computed PSC pharmaceutical properties included here may provide relevant insights into the rational drug design process, because, for example, it only takes a few minutes to screen 20.000 compounds using dry-lab tools such as those used here, but it would take several weeks in a wet-lab to obtain the same pharmaceutical profile. The knowledge gained with the PSC-db provides useful physicochemical and pharmaceutical information that could guide future drug development through medical chemistry optimization supported by computational tools such as virtual screening and molecular docking, even aided by machine and deep learning tools for large-scale in-silico screening [34]. The use of these molecular modeling techniques combined with chemical libraries such as the one presented in this study may help users to carefully select which PSC may be selected for further studies in drug discovery programs. It may also open new horizons in plant transportomics (academia, pharma, and agroindustry) because the PSC-db could be used to reverse-dock plant transporters and PSC to find new transporter-PSC pairs. Such approaches would significantly speed up the identification of the transport mechanisms of a specific phytochemical compound class by a selected plant transporter family.

## 4. Materials and Methods

PSC-db has been implemented using the open-source PostgreSQL database where all compounds with their complementary and calculated information (classification, redundant names, unique IDs, physicochemical properties, etc.) were hierarchically organized. In order to correctly extract the information deposited in this database, multiple sources were used and manually curated using in house scripts to verify the information. Among these sources are PubChem [35], KEGG [36], Japan Chemical Substance Dictionary Web (NIKKAJI), KNApSAcK [37], ChEMBL [38], and PDB-CCD [39] databases, used to connect and extract 2856 compounds of pharmaceutical and agro-industrial interest. These compounds were later processed through the SwissADME server [40] in order to obtain new relevant physicochemical properties. All original and calculated data were parsed and stored in our database using pipelines developed in python language. The web interface of PSC-db was developed using PHP, HTML, JavaScript, CSS, Bootstrap, jQuery, and AJAX technologies, and as user-utilities, the following characteristics were implemented: (a) interactive 3D-viewer, (b) online 2D-drawing tool for searching similar compounds by using the Tanimoto coefficient parameter as distance metric between a SMILES string from the molecule used in the search tool, and the molecules deposited in the PSC-db. The openbabel toolbox [41] and FP2 fingerprint were used for similarity search. (c) simultaneous and multiple filtering of the PSC search, (d) graphical generation of descriptors and relational statistics of the PSC properties, (e) determination of the r^2^ value of the correlation between any two physicochemical properties among a subset of compounds (this value is dynamically calculated on the client-side depending on the number of compounds selected using the Javascript programming language and its Math library), and (f) possibility to select and download all structural and physicochemical information of a particular subset of compounds defined by the user, as well as the entire database.

### 4.1. System Architecture

The architecture of the solution and the essential components are shown in Figure S5. This representation is divided into three main layers: the presentation, the domain, and the data layers. The presentation layer represents the user’s view and the interaction with the domain layer. It consists of two modules: (a) ParametersInput which is responsible for obtaining the necessary data to compute the request. These inputs can be text (total or partial compound name, formula), numbers (compound ID, min and max molecular weight) or chemical structures (using the molecular editor); (b) ResultsView, which is composed of the following modules:

i. HierarchicalView: performs a hierarchical representation of the compounds organized by subfamilies.
ii. TableView: shows the list of compounds as data tables. It is possible to filter the results in the data table writing some patterns into a text input. Also, a dynamic CSV or SDF file can be downloaded containing only the compounds listed (filtered) in the data table.
iii. DetailsView: allows access to detailed information of the compounds, such as external links, hierarchical organization, and its properties (physicochemical, lipophilicity, water-solubility, pharmacokinetics, and drug-likeness), source organism and biological activity.
iv. StatsView: generates statistics of the compounds and determines the correlation value (r2).
v. 2D/3DView: shows the 2D and 3D visualization of each chemical compound with its corresponding Radar ADME/Tox graph.

The domain layer represents the core of PSC-db and denotes the communication link between the presentation and data layer. This layer has the Core module, which is responsible for processing the user query, request the query to the databases (PostgresSQL or SDF) and returns the MOL2 and PNG files. The Core module also generates the temporal CSV and SDF files and prepares and returns the JSON files with the results.

The data layer has the following components which are responsible for data-storage: (a) PostgreSQL DB stores the information of compounds and its calculated properties; (b) SDF file of compounds contains all compounds; it is used to search for similar compounds (from the molecular editor); (c) JSON Files contains the processed results; (d) CSV Files is generated on the user’s request to download the filtered results (properties information of compounds); (e) SDF Files are generated on the user’s requests to download the filtered results (as 3D coordinates of compounds); (f) PNG files contains the 2D and Radar ADME/Tox images of compounds, (g) MOL2 Files contains the data for the interactive 3D viewer of the compound structures, and h) SQL files with database backup.

### 4.2. Database Model

The PostgreSQL database stores the data of every compound. The physical model of our database (Figure S6) has been designed according to the conceptual model including the following tables: (a) type stores the information corresponding to the two main subsets of compounds (general NPs and NPs used as drugs); (b) names contains all the different names of each compound; (c) class stores the different subset of classes to which each compound belongs; (d) compound stores all data of each compound. Among them are, for example, the smile format, the chemical formula, and all other 42 experimental and calculated physicochemical features (attributes); (e) comp_class is used to link each compound with its corresponding class; (f) ext_db stores the information of the external databases; (g) comp_ext connects each compound with the information stored in external databases; (h) target contains information about targets; (i) chembl stores the ChEMBL IDs; (j) target_type stores types of targets; (k) comp_tar stores references to source organisms; (l) family contains information about families; m) specie contains information about species; (n) kingdom contains information about kingdoms; and (o) comp_tax stores references to biological activities.

## Data Availability

PSC-db is freely available for public use at http://pscdb.appsbio.utalca.cl (accessed on 6 January 2021).

## Supplementary Materials

The following are available online, Figure S1: Illustrated record of PSC-db. Figure S2: Record for the L-Pipecolate alkaloid is shown, with the names and alternative denominations, as well as the external links (A) and hierarchical organization (B). Figure S3: Record for the L-Pipecolate alkaloid is shown with the physicochemical and pharmaceutical properties calculated with SwissADME server (A–E). Figure S4: Record for the L-Pipecolate alkaloid is shown with the source organism (A) and biological activity (B). Figure S5: The architecture of the solution and the essential components of PSC-db. Figure S6: Physical data model implemented on PSC-db.
