# Supplementary material for: PSC-db: A Structured and Searchable 3D-Database for Plant Secondary Compounds
Source: Molecules. 2021 Feb 20;26(4):1124. doi: 10.3390/molecules26041124 (PMC7924326; doi:10.3390/molecules26041124)
Supplement: Supplementary file 1 [file molecules-26-01124-s001.pdf]

PSC-db: A structured and searchable 3D-database for plant secondary compounds

Alejandro Valdés-Jiménez †, Carlos Peña-Varas †, Paola Borrego-Muñoz, Lily Arrue, Melissa Alegría-Arcos, Hussam Nour-Eldin, Ingo Dreyer, Gabriel Nuñez-Vivanco, David Ramírez*

* Correspondence: david.ramirez@uautonoma.cl; Tel.: +56 2 23036667
† These authors equally contributed to this work.

Supplemental information

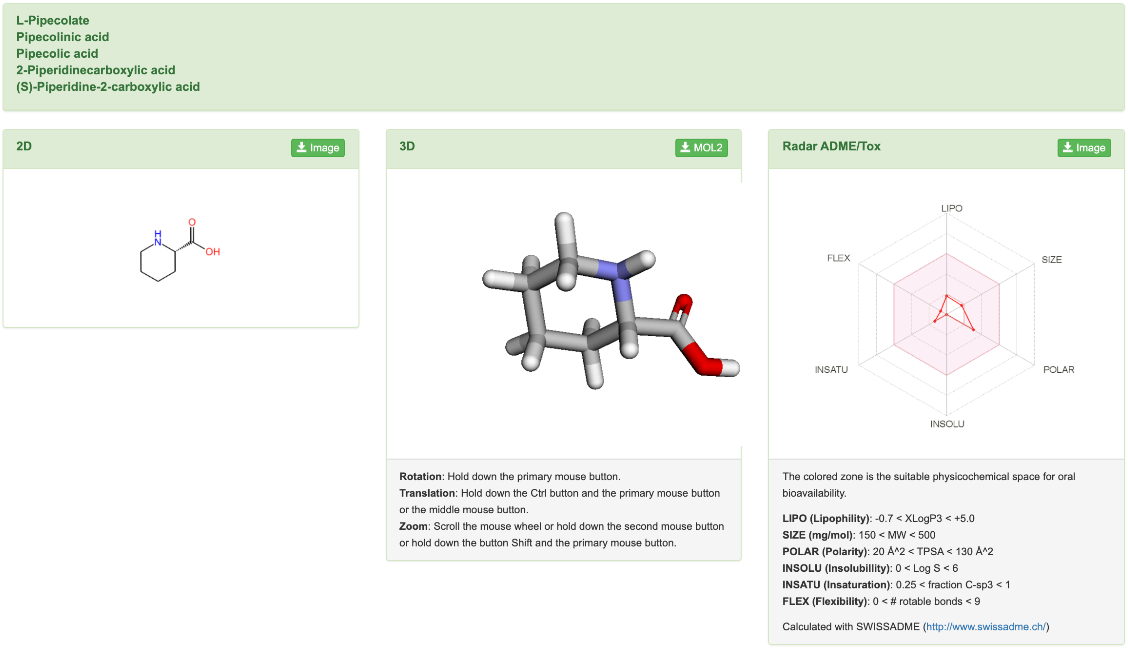

Figure S1. Illustrated record of PSC-db. A view of the 2D- and 3D-representation of a given molecule, as well as Bioavailability RADAR plotted with the predicted physicochemical and pharmaceutical features for the L-pipecolate alkaloid is shown.

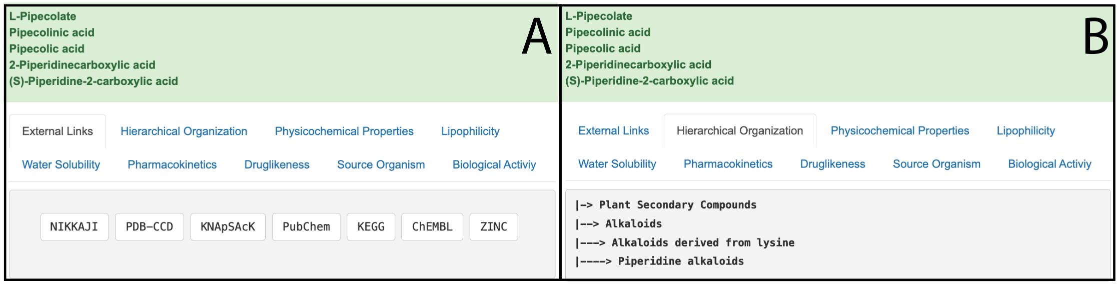

Figure S2. Record for the L-Pipecolate alkaloid is shown, with the names and alternative denominations, as well as the external links (A) and hierarchical organization (B).

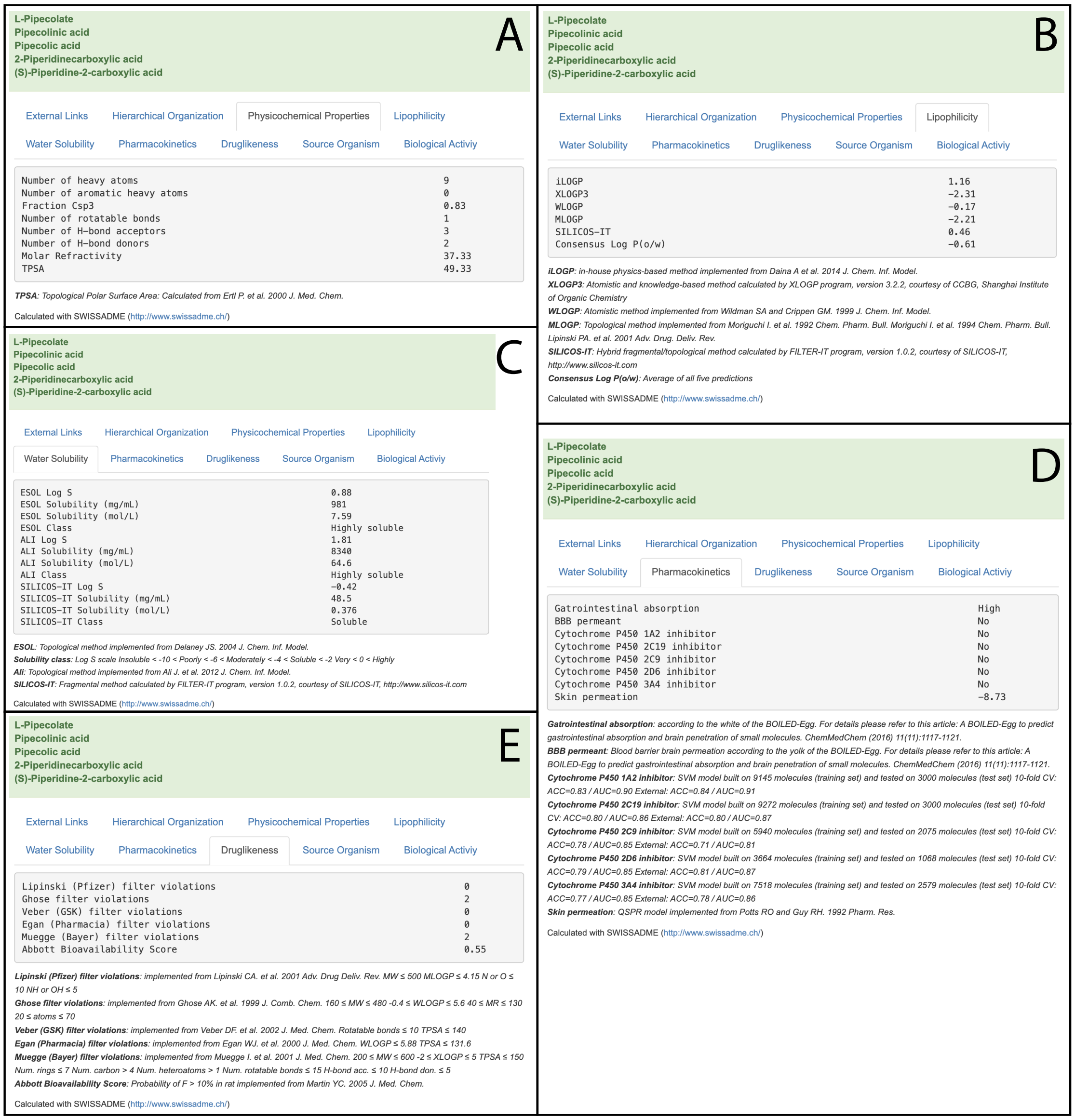

Figure S3. Record for the L-Pipecolate alkaloid is shown with the physicochemical and pharmaceutical properties calculated with SwissADME server (A-E).

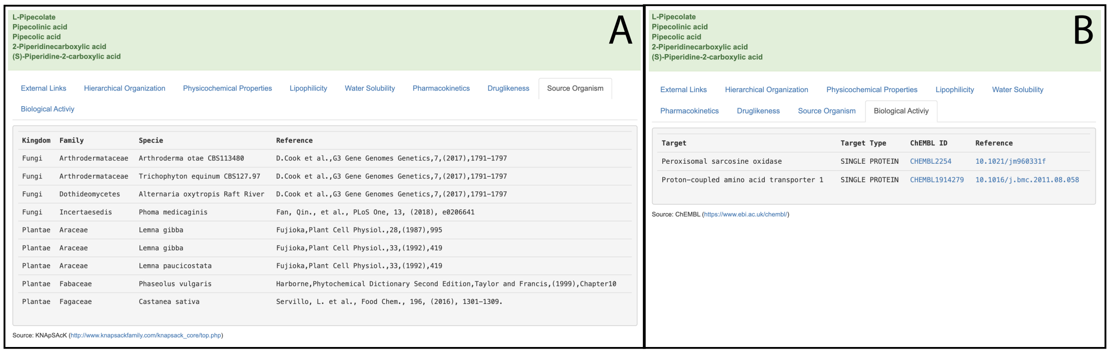

Figure S4. Record for the L-Pipecolate alkaloid is shown with the source organism (A) and biological activity (B).

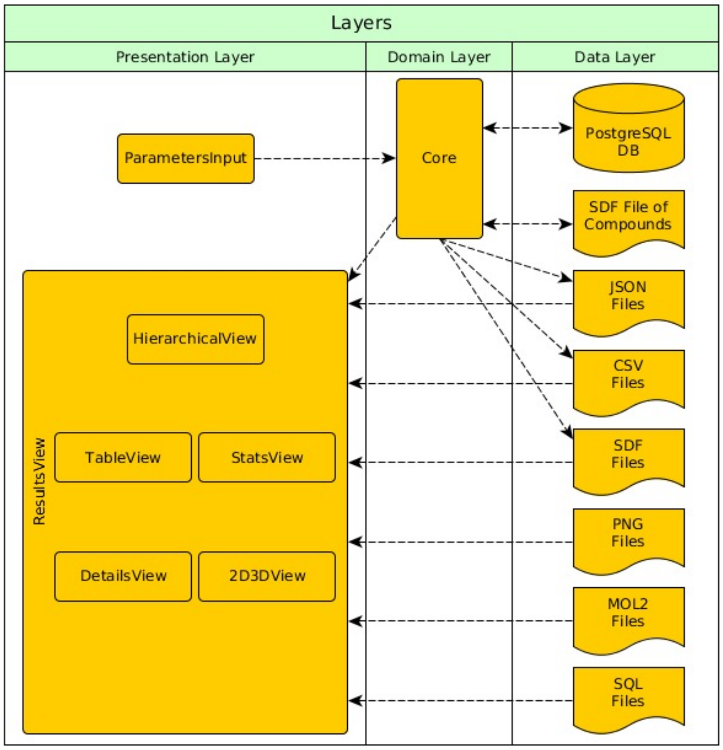

Figure S5. The architecture of the solution and the essential components of PSC-db. It consists of three main layers: the presentation, domain, and data layers, representing the interaction between the essential components of the solution.

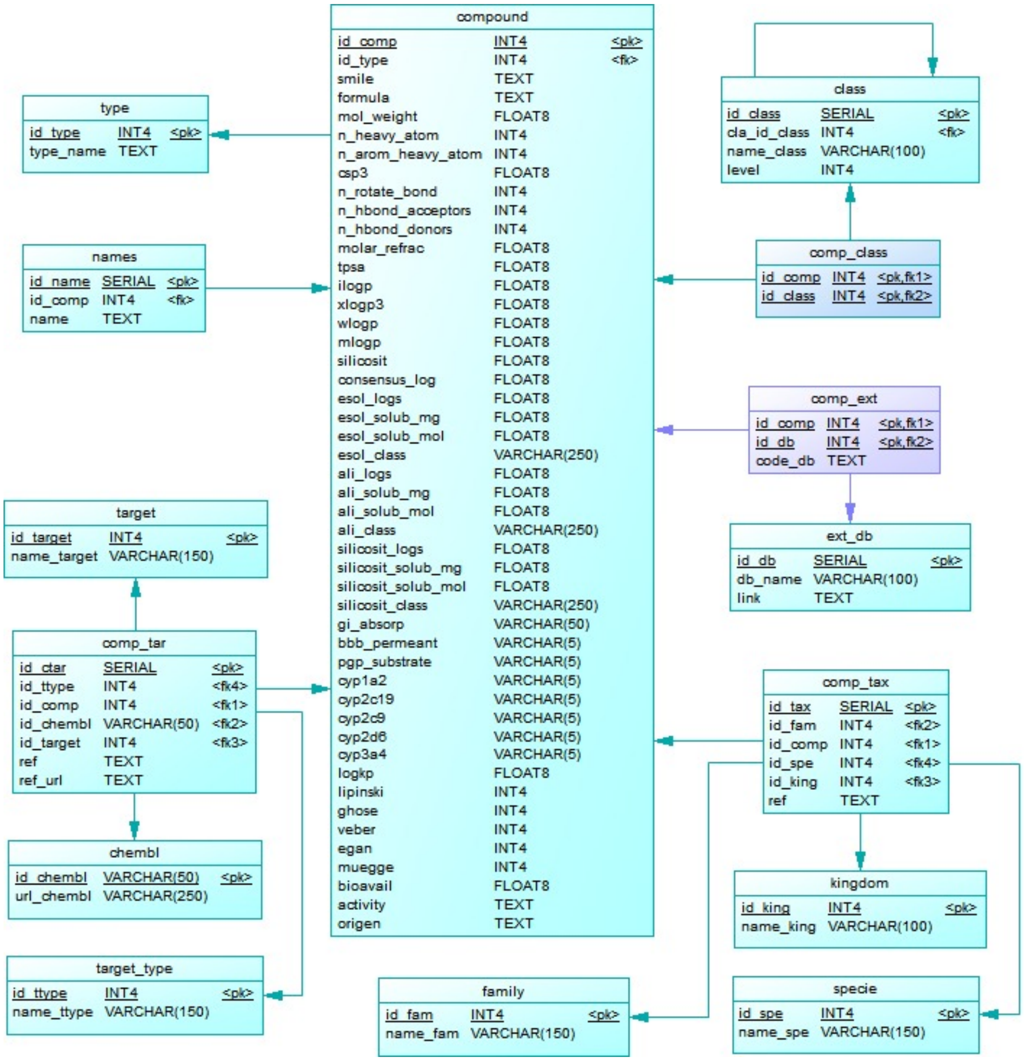

Figure S6. Physical data model implemented on PSC-db.
